# Periodontitis as a Risk Factor of Atherosclerosis

**DOI:** 10.1155/2014/636893

**Published:** 2014-03-23

**Authors:** Jirina Bartova, Pavla Sommerova, Yelena Lyuya-Mi, Jaroslav Mysak, Jarmila Prochazkova, Jana Duskova, Tatjana Janatova, Stepan Podzimek

**Affiliations:** Institute of Clinical and Experimental Dental Medicine, First Faculty of Medicine and General University Hospital, Charles University, Karlovo Namesti 32, 12000 Prague, Czech Republic

## Abstract

Over the last two decades, the amount of evidence corroborating an association between dental plaque bacteria and coronary diseases that develop as a result of atherosclerosis has increased. These findings have brought a new aspect to the etiology of the disease. There are several mechanisms by which dental plaque bacteria may initiate or worsen atherosclerotic processes: activation of innate immunity, bacteremia related to dental treatment, and direct involvement of mediators activated by dental plaque and involvement of cytokines and heat shock proteins from dental plaque bacteria. There are common predisposing factors which influence both periodontitis and atherosclerosis. Both diseases can be initiated in early childhood, although the first symptoms may not appear until adulthood. The formation of lipid stripes has been reported in 10-year-old children and the increased prevalence of obesity in children and adolescents is a risk factor contributing to lipid stripes development. Endothelium damage caused by the formation of lipid stripes in early childhood may lead to bacteria penetrating into blood circulation after oral cavity procedures for children as well as for patients with aggressive and chronic periodontitis.

## 1. Introduction

Epidemiological studies have established that periodontitis is a risk factor for cardiovascular diseases [1], lung diseases [2], renal diseases [3], and low birth weight in children [4]. Accordingly, it may be assumed that dental plaque bacteria not only influence the oral cavity locally, but may also contribute to the development of some serious systemic diseases.

The prevalence of cardiovascular diseases in patients with periodontitis is 25–50% higher than in healthy individuals. Poor self-reported oral health (as a possible risk factor for periodontitis) and tooth loss (as a possible consequence of periodontitis) are positively associated with a coronary atherosclerotic burden [5]. Severe tooth loss (likely to be due to periodontal disease) may be a predictor of cerebrovascular disease-silent cerebral infarct [6].

An association between oral health and cardiovascular disease has been proposed for more than a century. Recently, the possible links between periodontitis and atherosclerosis have intensified and are being investigated for possible association and causality. Common risk factors for these diseases include increasing age, smoking, alcohol abuse, ethnicity, educational and socioeconomic status, being male, diabetes mellitus, and obesity [7, 8]. Reviewed observational studies to date support an association between periodontitis and atherosclerosis but do not support a direct causative relationship. This extensive review illustrates an important general trend towards periodontal treatment-induced suppression of systemic inflammation and improvement in noninvasive markers of atherosclerosis and endothelial function [9].

In the late 1990s, periodontitis-atherosclerosis syndrome (PAS) was described and the number of articles devoted to PAS has increased every year. In 1998, there were only 4 articles on this subject, 73 articles in 2007 and at present there are 3928 articles focusing on PAS in the literature [10].

## 2. Periodontitis

Periodontitis is a chronic inflammatory disease that affects the tooth supporting tissue—the periodontium. It is the most frequent cause of tooth loss in the adult population. The prevalence of the disease is high, with the moderate form affecting 50% and the progressive form 5–15% of the adult population [11]. Periodontitis is a multifactorial disease and as such the significant elements are not only the presence of pathogenic bacteria and the immune mechanism, but also the genetic predisposition of the patient. The origin and progress of the inflammatory reaction in the periodontium are a result of the altered interplay of the defense mechanisms in the periodontal tissue to respond to the activity of dental plaque bacteria.

The causes of the onset and progress of periodontitis have been investigated for hundreds of years. The first records concerning the disease now called periodontitis date back to the ninth and tenth centuries A.D. with Arabian physicians already ascribing the disease to soft plaque on teeth. The assumption that dental plaque was one of the significant etiological factors was confirmed as recently as the 1960s [12, 13]. During this time, the first articles appeared in which the authors demonstrated that patients' blood serum had enhanced levels of antigens reacting with dental plaque bacteria [14].

A typical symptom of periodontitis is the periodontal pocket. A low redox potential, supply of nutrients in the crevicular fluid, and limited amount of oxygen in the periodontal pocket characterize the optimal conditions for the occurrence of Gram-negative anaerobic bacteria. Among the bacteria involved in pathogenesis of the disease are Porphyromonas gingivalis, Prevotella intermedia, Fusobacterium nucleatum, Tannerella forsythia, Treponema denticola, and others. Most of these pathogenic bacteria belong to Gram-negative bacteria that contain the lipopolysaccharides (LPS), a potent activator of B lymphocytes. Porphyromonas gingivalis is one of the most important pathogenic bacteria due to the production of a protease which breaks and deactivates IL-1beta. It additionally contains a cysteine protease called gingipain which is specifically split by CD14 molecule, a receptor for LPS. This enzyme enables the bacteria to suppress the immune reaction against LPS [15]. Although the presence of subgingival microbiota is a necessary condition for the disease to progress, it is not the only cause. A genetically dependent effect in the immune mechanism or a modified immune reaction on the presence of pathogenic bacteria may be also involved in disease progression.

## 3. Atherosclerosis

The amount of evidence corroborating the association between specific bacteria and coronary diseases developing as a result of atherosclerosis has increased over the last two decades. These findings have brought a new aspect to the etiology of the disease. The original classical hypotheses about the development of atherosclerosis did not include inflammation as a primary factor of the pathogenesis. One of the hypotheses assumed that changes of endothelium as a consequence of mechanical damage with subsequent contamination by toxins, metal ions, and free radicals lead to the formation of atherosclerotic plates [16–19]. Another hypothesis assumed that lipoproteins of low density caused the atherogenesis, transported by endothelium to the intima, where they oxidize and act as chemoattractant of monocytes/macrophages, leading to the formation of foam cells [20–24]. In contrast, other studies [25, 26] have illustrated the significance of proliferation of smooth muscles in the intima.

In the last decade, it has been demonstrated that atherosclerosis begins as an inflammatory reaction against endothelial cells and other components of the artery wall. The inflammation sites attract accumulation of macrophages, T and B lymphocytes, and mast cells. The blood vessel walls are also covered by deposited fats which led to occlusion of the vessels. Atherosclerosis is however a multifactorial disease. Among the risk factors are circulating lipoproteins (hypercholesterolemia), genetic predisposition, hypertension, smoking, obesity, and diabetes. Epidemiological studies further indicate that infection by various types of bacteria, including periodontopathic ones (Chlamydia pneumoniae, Helicobacter pylori, Porphyromonas gingivalis, Prevotella intermedia, and Aggregatibacter actinomycetemcomitans) and the presence of products of these bacteria (LPS, heat shock protein (HSP)) in serum contributed to the development of atherosclerosis [27]. LPS stimulates monocytes/macrophages by binding to the CD14 as the receptor. A genetically conditioned reaction to bacterial stimulation may play a certain role in pathogenesis of atherosclerosis. Patients who survived myocardial infarction exhibited a higher frequency of allele T(-260) in the promoter of gene for the CD14 receptor than controls [28].

Patients with verified atherosclerotic changes further exhibited enhanced serum levels of antibodies against HSP 60 [29]. Experiments performed on animal models show that infection may lead to enhanced induction of antibodies against HSP 60. These antibodies may bond to HSP 60 expressed on endothelium of blood vessels at sites of their bifurcation where the endothelium is in stress. The binding of antibodies to the endothelium surface may be the triggering mechanism of inflammatory autoimmune disease.

A recent review [30] dealt with the epidemiological and etiopathogenetic association between chronic periodontitis and stroke. It specifically reviewed the relationship between oral infection caused by dental plaque bacteria and the stimulation of proatherogenetic mechanisms as atherosclerosis of the cerebral vessels and ischemic stroke are most frequent causes of acute stroke.

## 4. Dental Plaque Bacteria and Atherosclerotic Processes

There are several mechanisms by which dental plaque bacteria may initiate or worsen atherosclerotic processes:

- activation of innate immunity,
- bacteremia related to dental treatment,
- direct involvement of mediators activated by dental plaque antigens in atheroma processes,
- involvement of cytokines and heat shock proteins from dental plaque bacteria,
- common predisposing factors influencing both diseases.

### 4.1. Activation of Innate Immunity

The oral cavity is permanently exposed to the activity of bacteria colonizing it. The epithelium forms not only a physiological barrier but also interacts with an innate immune response resulting in the production of antimicrobial peptides. Important components of innate immunity at sites of contact with microorganisms are alkaline antimicrobial peptides that contain less than 100 amino acids and are phylogenetically very stable; they exist in both plant and animal life. In mammals, they are present in phagocyte granules, are produced by the epithelium, and are present in bodily fluids and secretions; the most important are defensins and cathelicidins. These antibacterial peptides kill various microorganisms and some of them are chemotactic.

Of particular importance for defense in the oral cavity are *β* defensins, calprotectin, histatin and, only in humans, cathelicidin-LL37/hCAP18 [31]. Defensins and histatin present in the phagocyte granules are produced by mucosal epithelium and by salivary glands. Cathelicidin is produced by phagocytes, the epithelium and salivary glands. It binds LPS, neutralizes endotoxine activity and acts chemotactically on neutrophils, monocytes, T lymphocytes, and mast cells and exhibits bactericidal activity. Its presence in large quantities in the junctional epithelium as a result of the migration of neutrophils is of great importance for the defense of the oral cavity [32]. Functional defects of neutrophils are a risk factor of the development of aggressive periodontitis [33, 34].

Both the Gram-positive and Gram-negative bacteria of dental plaque contain many structural and secretory components that either directly damage the periodontal tissue or stimulate the immune system of the host.

The cell walls of Gram-negative bacteria are formed of peptidoglycans, polysaccharides, proteins, lipids, lipopolysaccharides, and lipoproteins [35]. The walls of Gram-positive bacteria consist of peptidoglycans, teichoic acid, and polysaccharides. LPS influences the immune reaction by binding to Toll-like receptor-4 (LPS of Escherichia coli and Aggregatibacter actinomycetemcomitans) or to Toll-like receptor-2 (LPS of Porphyromonas gingivalis). LPS also stimulates expression of costimulatory molecules CD80/CD86, via binding to Toll-like receptor-4; furthermore, it stimulates molecules of the major histocompatibility complex MHC-II which are important for activation of T-cells.

Peptidoglycans activate the cells through binding to the Toll-like receptor-2; they are recognized by the complement as well as by specific receptors [36] and they also participate in the activation of the complement system.

The immune response, directed against an infection, also leads to further destruction of the tissue [29]. It was confirmed in* in vitro* experiments that cells of the junctional epithelium activated by Porphyromonas gingivalis produce TNF-*α* and IL-1*β* and express surface molecules ICAM-1 and VCAM-1 [37]. The influence of oral bacteria on the cytokine network is more complicated. It was shown that Porphyromonas gingivalis inhibits accumulation of IL-8 in gingival epithelium cells [38]. Porphyromonas gingivalis also produces proteases which cleave and inactivate IL-1*β* and the cysteine protease-gingipain, which specifically cleaves CD14 (receptor for LPS). This enzyme enables the bacteria to suppress the immune response to LPS [15]. This mechanism is known as “localized chemokine paralysis.” Gingipain produced by Porphyromonas gingivalis degrades proteins to generate free arginine or lysine; the primary goal of the degradation is to obtain the peptides and amino acids necessary for survival of the bacteria. However, it also degrades many important molecules on the surface of cells or in its environment and thus it protects P. gingivalis against the immune reaction. This enzyme also degrades IL-8, IL-1*β*, IL-6 [39, 40], surface molecules ICAM-1 on epithelium cells [41], CD14, lipopolysaccharide binding protein (LBP), molecules on the surface of monocytes and fibroblasts [42], components of the complement, and also immunoglobulins [43].

Among the markers of developing inflammation are enhanced levels of C-reactive protein (CRP) in serum. CRP belongs to the highly conservative pentraxin family of proteins significant to the innate immune reaction. CRP is bound to apoptotic cells, oxidized low density lipoprotein (ox-LDL) and oxidized phospholipids, but do not bind to native low density lipoprotein. It is assumed that CRP is involved in modulation of developing atherosclerosis, because CRP and ox-LDL are present in atherosclerotic lesions. Slightly enhanced concentrations of CRP may predict coronary disease [44–46].

Another sign of an activated innate immune system is an enhanced level of neopterin in the patient's serum. A high concentration of neopterin corresponds to a high degree of activation of the immune reaction in acute coronary syndrome [47].

Bacteria of dental plaque and their components in the periodontal tissues may penetrate into the circulation system and exhibit pathogenic potential.

### 4.2. Bacteremia Related to Dental Treatment

Increased incidence of bacteremia by Gram-negative bacteria and infectious endocarditis was described more than 30 years ago.

This synoptic review [48] summarizes the cases of infectious endocarditis caused by bacteria of dental plaque. The following bacteria were proven as etiological factors: Aggregatibacter actinomycetemcomitans, Eikenella corrodens, Streptococcus species, Capnocytophaga, Neisseria, and Lactobacillus.

Dental infection affecting the periodontium can spread into the systemic circulation by dental treatment procedures or teeth brushing and can induce bacteremia. Patients with untreated adult periodontitis are at greater risk of bacteremia after periodontal probing than patients with chronic gingivitis [49]. The predominant microorganism of dental plaque—Streptococcus sanguis—is associated with endocarditis [50]. Following dental extraction, the most frequently identified bacteria in the positive blood cultures were the Streptococcus species. Positive blood cultures persisted for 1 h after dental treatment procedure [51]. High incidence of bacteremia was found in patients without antibiotic prophylaxis after conservative and surgical dental treatment [52]. Low incidence of bacteremia was demonstrated after orthodontic banding and debanding [53, 54]. Bacteremia following periodontal procedures was also described [55]. Dental surgical procedures were a cause of bacterial endocarditis in children. In these cases, viridans streptococci were mainly detected [56, 57]. In another study [58], an increased level of dental plaque bacteria in blood circulation after dental plaque removal and tooth extraction were described. On the other hand, chewing did not cause bacteremia in chronic periodontitis patients [59].

Endotoxins are capable of generating a range of systemic and local host responses [60]. No effects on the incidence of bacteremia were found after subgingival irrigation [61].

### 4.3. Direct Involvement of Mediators Activated by Dental Plaque Antigens in Atheroma Processes

The ability of Porphyromonas gingivalis to actively invade aortic and heart endothelial cells is an example of the relationship between periodontitis and atherosclerosis.

The presence of Porphyromonas gingivalis and Streptococcus sanguis in atherosclerotic plaques in samples of veins after surgical reconstruction of venous system was established [62]. Using PCR reaction, microbial ribosomal RNA (rRNA) and DNA from Porphyromonas gingivalis, Aggregatibacter actinomycetemcomitans, and Prevotella intermedia were detected in atherosclerotic plaques [63–66]. A possible link between periodontal disease and abdominal aortic aneurysm was examined. Resected specimens from abdominal aortic aneurysm were positive for periodontal bacterial DNA in 86% of cases. The presence of bacteria was demonstrated in the intima layer of the atherosclerotic occlusive aorta but not in control specimens [67].

In 31 carotid endarterectomy specimens, Porphyromonas gingivalis was detected in 52%, Fusobacterium nucleatum in 34%, Tannerella forsythia in 34%, Prevotella intermedia in 41%, and Aggregatibacter actinomycetemcomitans in 17% [68]. In a recent review [69], the results of 16 studies investigating the presence of oral bacteria in atheromatous plaque were compared, identifying Aggregatibacter actinomycetemcomitans and Porphyromonas gingivalis as the most frequently occurring bacteria.

The relationship between atherosclerosis and Porphyromonas gingivalis was confirmed experimentally using the model of Apo E-null mice. The development of periodontitis and atherosclerosis was induced by oral inoculation by Porphyromonas gingivalis. Over the course of 4 months, the mice exhibited lipid stripes in which the presence of Porphyromonas gingivalis was detected, and in mice with periodontitis, higher serum levels of IL-6 and VCAM-1 in aorta were detected [70].

Recent studies show that inflammation may be supported by the presence of periodontopathogenic bacteria. Study on the influence of microbiota showed that both conventional and germ-free kept mice with ApoE*‒*/*‒* deficit on a high cholesterol diet had lesions of heavy atherosclerosis in thoracic and ventral aortas. In some cases, these lesions completely obstructed the vessel. In germ-free mice on a diet with high cholesterol content, histopathological evaluation of removed tissue samples displayed greater damage of organs than that found in conventionally kept mice. The authors confirmed the importance of the conventional microbiota for the protection of tissues [71].

### 4.4. Involvement of Cytokines and Heat Shock Proteins from Dental Plaque Bacteria

After stimulation by bacteria and their components (LPS, peptidoglycans), the periodontal tissue produces inflammatory cytokines (IL-1*β*, TNF-*α*, IL-6, INF-*γ*, IL-12, IL-10), chemokines (MCP-5, IL-8, MIP-1*α*), prostaglandine PGE_2,_ and NO [72, 73]. LPS from Aggregatibacter actinomycetemcomitans significantly enhances expression of **β**_*2*_ integrins and L-selectins.

Peptidoglycans are components of the bacterial walls [35] and, like LPS, they contribute to activation of immune cells via binding to TLR-2 receptor. In addition, peptidoglycans are recognized by the complement system and specific receptors resulting in production of TNF-*α*, IL-1*β*, IL-6, IL-8, and MIP-1*α* [74, 75] and NO in macrophages [76]. In comparison with LPS, peptidoglycans are not so strong stimulators of immune reaction.

The presence of circulating oral bacteria or bacterial components may stimulate blood cells to produce cytokines. IL-6 levels significantly increased eight hours after scaling, while IL-8 decreased [77]. Higher levels of IL-6 were detected in the sera of patients with periodontitis compared to healthy controls [78].

Heat shock proteins (HSPs) are known to be the most immunogenic antigens of bacteria. The extensive homology between human and bacterial HSPs may play a role in the activation of atherosclerotic changes. Decreased proliferative responses of peripheral blood cells to HSP in periodontitis patients compared to control patients were found [79].

Decreased production of IFN-*γ* after stimulation of peripheral blood mononuclear cells with HSP 60 and HSP 70 was observed in periodontitis patients as compared to control patients. These findings support the hypothesis of suppressed Th1 response in periodontitis patients that may lead to increased susceptibility for development of aggressive periodontitis. Antibodies against human HSP 60 and antibodies against Porphyromonas gingivalis (GroEL) in sera and inflamed gingival tissues were found in periodontitis patients [80]. A quantitative analysis of serum antibodies demonstrated significantly increased levels in periodontitis patients as compared to controls. Anti-Porphyromonas gingivalis GroEL antibodies were detected in all samples of inflamed gingival tissues of periodontitis patients. Enhanced levels of antibodies against HSP 60 were also found in the serum of patients with positive atherosclerotic changes [29]. Molecular mimicry between GroEL of the periodontopathic Porphyromonas gingivalis and autologous human HSP 60 may play a role in immune mechanisms. Experiments performed on animal models show that bacterial infection may lead to enhanced production of antibodies against HSP 60 expressed on the endothelium of blood vessels at sites of their bifurcation where the endothelium is in stress. The binding of antibodies to the endothelium surface may be a triggering mechanism of inflammatory autoimmunity disease [81]. Several studies have demonstrated that the immune response to HSP 60 may be involved in the pathogenesis of both atherosclerosis and chronic periodontitis. Antibody levels to human as well as to Porphyromonas gingivalis HSP 60s were the highest in patients with atherosclerosis in comparison to healthy controls. Clonal analysis of the T cells clearly demonstrated the presence of both human and Porphyromonas gingivalis HSP GroEL-reactive T-cells in the peripheral circulation of patients with atherosclerosis. These results suggest that T-cell clones with the same specificity may be involved in the pathogenesis of the different diseases [82]. Analysis of the nucleotide sequences of the T-cell receptor (TCR) demonstrated that human HSP 60-reactive T-cell clones and T-cells have the same receptors infiltrating periodontitis lesions [83]. Analysis of the cytokine profile demonstrated that HSP 60-reactive peripheral blood mononuclear cells produced significant levels of IFN-*γ* in periodontitis patients, whereas Porphyromonas gingivalis GroEL did not induce type 1 or type 2 cytokine profiles. In control subjects, no significantly increased expression of IFN-*γ* or IL-4 was induced. These results suggest that periodontitis patients have human HSP 60-reactive T-cells with a type 1 cytokine profile [82]. In another study [66], GroEL specific T-cell lines from peripheral blood and GroEL human HSP 60 and Porphyromonas gingivalis specific T-cell lines from atherosclerotic plaques were characterized in their cross-reactivity. The cytokine profiles of arterial T-cell lines specific for GroEL, human HSP 60, and Porphyromonas gingivalis were Th2 CD4^+^ cells predominantly. Cross-reactivity between bacterial cells, including periodontal pathogens, with endothelial cells expressing HSP 60 may explain an association between atherosclerosis and periodontal disease [66].

The nature of inflammatory infiltrate and the presence of HSP and GroEL were examined in 31 carotid endarterectomy specimens. Human HSP 60 was detected on surface of endothelial cells, smooth muscle cells, and lymphocytes; GroEL and bacteria were detected within intimal cells [68].

Endogenous HSP is also a target of autoantibodies in autoimmune disorders, atherosclerosis and vascular diseases. HSP is one of the endothelial cell autoantigens able to trigger cytotoxic and apoptotic response by related autoantibodies [84, 85].

Marked atherogenic effects of repeated immunizations with Porphyromonas gingivalis, bacterial and host HSP, were examined in ApoE−/− deficient mice. The development of lesions in proximal aorta correlated with the levels of HSP 60 and GroEL antibodies and may be explained as molecular mimicry between GroEL and HSP 60 in Porphyromonas gingivalis immunized ApoE−/− mice [86].

The presence of Porphyromonas gingivalis and Tannerella forsythia increased in patients with myocardial infarction and periodontitis with periodontal pockets deeper than 4 mm. The authors of this study also reported a correlation between these bacteria and increased levels of corresponding antibodies against HSP 60 in patients' sera [87].

### 4.5. Common Predisposing Factors Influencing Both Diseases

Several risk factors are common for both diseases. They include smoking, obesity, and diabetes [88]. Long-term studies indicate that patients with periodontitis have a 20–25% higher risk of myocardial infarction [89, 90] and a 17% higher risk of brain stroke [91]. Both diseases are multifactorial and start in adolescence rather than in adulthood. The first lipid stripes can appear in blood vessels of 10-year-old children [92], and foam cells have been found in children less than 1 year old [93]. It remains an open question whether periodontitis is the cause or a supporting factor of atherosclerosis [94].

## 5. Conclusion

The relationship between periodontitis and atherosclerosis has been a subject of many research activities with the number of publications focusing on this relationship rapidly increasing in recent years. The actual number of studies which focus on this subject is 3928 according to a review from this year [10].

Periodontitis and atherosclerosis are multifactorial diseases with an onset in early childhood, although first symptoms may appear in adulthood.

Since foam cells have been found in early childhood [93] and lipid stripes in 10-year-old children [92], we do not think that in these cases, periodontitis is the cause of changes in blood vessels. Increased prevalence of obesity in children and young people is a risk factor that may influence lipid stripes development [95, 96]. However, endothelial damage by formation of lipid stripes in early childhood may lead to the capture of bacteria of dental plaque origin that penetrate into blood circulation after treatment procedures in oral cavity of children as well as of patients with aggressive or chronic periodontitis. Preatheroma and atheroma are usually diagnosed in patients aged 20–30 years—similar to the age which aggressive periodontitis (early onset periodontitis) is diagnosed. Fibroatheroma is diagnosed in patients aged 40 years and over, in the similar age group where periodontitis is diagnosed in more than 50% of patients.

When solving the question whether periodontitis is the cause or consequence of developing atherosclerosis, we may conclude that circulating microorganisms or their products (HSP) may promote pathogenesis and enhance local inflammatory changes in vessel walls that may promote clotting and clot formation. Dental plague bacteria are one of the risk factors for atherosclerosis development [69].

The concept of risk factors is the basis of the preventive approach in medicine. Prevention programs focused on monitoring patients with chronic periodontitis in relation to the risk of developing cardiovascular diseases should be emphasized. In patients with early inflammatory symptoms in the periodontium, a dental examination should be supplemented with a laboratory examination of cardiovascular markers. Timely therapy of both diseases diminishes the risk of developing initial as well as serious changes at a later time.

Since bacteremia develops after dental treatment and the first lipid stripes arise in early childhood, research of early phases of atherosclerosis and a more precise specification of the risk factors of the disease could lead to an enhanced quality of preventive care and treatment of patients at a later age. Antibodies in the patients' sera reacting with ox-LDL and HSP 60 and HSP 65 may be detected in very young patients with aggressive periodontitis and may be a predicative factor in the development of serious systemic diseases [92].
